# Is low health literacy associated with overweight and obesity in adolescents: an epidemiology study in a 12–16 years old population, Nanning, China, 2012

**DOI:** 10.1186/2049-3258-72-11

**Published:** 2014-04-01

**Authors:** Lawrence T Lam, Li Yang

**Affiliations:** 1 Discipline of Paediatrics and Child Health, Sydney Medical School, The University of Sydney, Sydney, Australia; 2 Department of Health and Physical Education, The Hong Kong Institute of Education, Hong Kong SAR, Hong Kong, China; 3 School of Public Health, Guangxi Medical University, Nanning, Guangxi, China

**Keywords:** Health literacy, Overweight, Obesity, Adolescents, Epidemiology study

## Abstract

**Background:**

The problem of overweight and obesity in children and adolescents is considered an epidemic in both developed and developing world by the WHO. There has been little study on the relationship between health literacy and body weight among adolescents.

This epidemiological study aims to investigate the association between low health literacy and overweight and obesity among a population of Chinese adolescents aged 12–16 years in the city of Nanning, China in 2012.

**Methods:**

This study was a population-based cross-sectional health survey utilising a two-stage random cluster sampling design. The sample consisted of high school students aged between 12–16 years with the total student population attending high schools in a large city as the sample frame. Health literacy was measured by the Chinese version of the short form of the Test of Functional Health Literacy translated for and validated among Taiwanese adolescents. Overweight and obesity were assessed in accordance to the recommendation of the World Health Organization (WHO) Global Database of Body Mass Index classification methods. Data were analysed using logistic regression modelling techniques with adjustment to the cluster sampling effect.

**Results:**

A total of 1035 students responded to the survey providing usable information with 628 (48.1%) respondents classified as high, 558 (42.8%) moderate, and 119 (9.1%) low levels of health literacy. After adjusting for potential confounding factors and the cluster sampling effect, low health literacy was significantly associated with overweight and obesity (OR = 1.84, 95% C.I. = 1.13-2.99).

**Conclusion:**

Results suggested that low health literacy level was associated with many aspects of adolescence health including their body weight. These results have public health implications on an important global problem of adolescence body weight. Enhancing the health literacy should be considered as part of the strategies in combating adolescence weight problem.

## Background

The US Institute of Medicine (IoM) first defined health literacy as: “the degree to which individuals have the capacity to obtain, process and understand basic health information and services needed to make appropriate health decisions” [1]. WHO further enhanced this definition to “the cognitive and social skills which determine the motivation and ability of individuals to gain access to, understand and use information in ways which promote and maintain good health” [2]. These represent two slightly different conceptual formulations of health literacy with the former focuses on the individual abilities of literacy and numeracy skills essential to understand health information, whereas the latter concentrates on the utilisation of skills that are crucial for an individual in interacting with the health system [3,4].

Different instruments have been developed aiming to measure health literacy in the population [5-16]. However, their quality has been questioned [17]. As a result different instruments have been used in different health literacy studies rendering an accurate estimate of population health literacy levels difficult. Despite the lack of a uniform assessment of health literacy in the general population, results of an earlier report indicated that a large proportion of the population had inadequate health literacy levels, particularly the older age groups. Nearly two thirds of Americans aged 60 years or older had poor health literacy skills and the majority (81%) of older patients could not understand basic medical information such as prescription labels [18]. Based on the available data in 2007, a review study on health literacy among geriatric patients found that the health literacy level of older patients within the population of many countries was low [19]. A more recent study conducted in Ireland using the short form of the Test of Functional Health Literacy in Adult (s-TOFHLA) found that about 14% of adults in the community were classified with limited health literacy [20]. Due to the lack of global studies on health literacy specifically conducted among adolescents, little information can be obtained on the overall health literacy levels of young people in different regions of the world, not to mention any regional comparisons. A recent study among high school students in Texas US reported that slightly more than half (52%) had adequate level of health literacy [21]. In the East Asia region, Chang translated and validated a short version of the s-Test of Functional Health Literacy (TOFHLA) into the Chinese language to be used among adolescents [22]. A study was subsequently conducted to investigate the health literacy level of young people in Taiwan and found that nearly 41% could be classified as high, 49% moderate, and nearly 10% low [23].

Bodyweight has become an important topic in adolescence health in recent years [24]. Particularly, the problem of overweight and obesity in children and adolescents is considered an epidemic in both developed and developing world by the WHO [25]. In terms of the relationship between general health literacy and bodyweight, particularly overweight and obesity in children and adolescents, not many studies have been conducted in the past three decades. In an earlier comprehensive systematic review of the literature on health literacy and health outcomes for all ages by DeWait et al. in 2004, no studies on the relationship between health literacy and overweight or obesity were found [26]. In a more recent update systematic review of literature since 2003 by the same research team, a few more studies were found on the relationship between health literacy and body weight and obesity [27]. The results suggested that most studies were flawed with methodological problems, particularly few had included potential confounding factors to be controlled for in the examination of the relationship [27]. Moreover, some of these studies were not focusing on the health literacy children or adolescents and their body weight, but the health literacy levels of the mother or the study was conducted in a specific patient group such as overweight children and not in the community [28,29]. In the small scale study by Sharif and Blank, it was found that child health literacy correlated negatively with the Body Mass Index (BMI) with a correlation coefficient of -0.37 (p < 0.001) among 78 overweight children [29]. However, this study only investigated the relationship between health literacy and body weight in a small group of children who were clinically diagnosed with obesity. In terms of studies on the association between general health literacy and bodyweight, particularly being overweight and obese in a non-clinical adolescence population none have been found in the literature.

This study aims to bridge the knowledge gap in examining the relationships between low health literacy and overweight and obesity in a Chinese adolescence population in the community.

## Methods

This study was a population-based cross-sectional health survey utilising a two-stage random cluster sampling design. The study was conducted in rural areas of the Nanning city in the Guangxi Province of the South Western region of China in November 2012. Nanning, the capital city of the Guangxi Province, is the biggest and most populated city of the Province with an estimated population of about 6.7 million in 2010. The population size for young adolescents aged between 15 and 19 years was estimated to be 505677. This represented about 7.6% of the total population in the city. Institute ethics approval for the study was granted by the Human Ethics Committee of the Hong Kong Institute of Education (RG46/12-13R).

The sample consisted of high school students aged between 12–16 years with the total student population attending high schools in the designated region as the sample frame. The local education department provided a list of high schools located within the boundary of the school district for sampling. The sample was generated using a two-stage random cluster sampling technique. First, using individual schools as the primary sampling unit, a number of schools were randomly selected with a probability proportional to the size of the target population. Second, using the class as the secondary sampling unit, different clusters of students were randomly selected from each grade of the selected schools. Participates were recruited from four high schools and 36 different classes.

The health survey was conducted within two weeks on campus at different schools. Students and their parents of the selected classes from different schools were informed of the survey with a written information letter. They were invited to participate in the study and according to the local regulations stipulated by the Educational Bureau of the Guangxi government willful consent was implied by the filling in of the questionnaire. During the survey students were asked to fill in a self-reported questionnaire designed specifically for the study.

Health literacy was measured by the TOFHLA which was originally designed by Parker et al. based on the Modified Cloze procedure for the adult population and the short version of TOFHL (sTOFHL) was developed by the same team [6,10]. The TOFHLA was further adopted and modified to be used for adolescents by Chisolm and Buchanan from the original adult version of the TOFHL [16]. Results of the Chisolm and Buchanan study supported the use of the reading and comprehensive component of the adult TOFHL to be used in the adolescent population, whereas the numeric component was not recommended owing to the lack of convergent validity. Based on the development theories in adolescent, it was further argued that the numeric component could be considered less important in comparison to the reading and comprehension component [16]. Hence, the TOFHLA was then adopted to be the TOFHL for adolescents. In terms of the Chinese version of the TOFHL, it was translated from the sTOFHL, validated among Taiwanese adolescents, and named as the sTOFHL for adolescents (sTOFHLA) by Chang et al. [22]. It consists of 36 items in a series of two health-related reading passages. In each passage, words are deleted from the original text and respondents must fill in the blank by selecting an appropriate word from a list of four possible choices. Results from a validation study suggested good reliability and validity. Confirmatory Factor Analysis confirmed a 2-factor structure fitted well with the data with satisfactory goodness-of-fit statistics. Cronhach’s alpha values provided evidence for good internal reliability with a value of 0.85. Test-retest coefficients for one week time period fell was 0.95. Convergent and discriminant validity were also demonstrated through predicted correlations with other self-report well-being indexes. For ease of analysis, the raw scores were categorised into three levels of health literacy, low, moderate, and high, in accordance to the cut-off points of <25, 25–31, and 32 or above as suggested by Chang [23]. The reasons for using the TOFHLA as the assessment instrument of health literacy in this study were two: first, this was the only translated and validated instrument suitable for use among Chinese youth; second, for the target age group of 12–16 years it would be more appropriate to apply a health literacy concept that relates closer to the health experience and needs of the target population such as be able to understand medical information.

Information collected in the survey included demographics, whether the respondent was a single child, parental education levels, whether the respondent was living with parents, ownership of a computer, and school performance with a total of 100 marks scaled in 10 marks interval during regression analysis. Data on health risky behaviours, including drinking, smoking, and sedentary behaviour of respondents were recorded using a frequency and duration exposure methods for quantify moderately rigorous activity involvement. Adopting the WHO recommended level of 60 minutes of moderate-to-vigorous daily physical activity for young people aged 5–17 years, a level less than half of the recommended level was considered as physically inactive in this study [30]. This was based on the assumption less than half of the duration of physical activity could not provide sufficient health benefit Information on drinking and smoking was collected using standardised and validated instruments. Familial information was also collected including respondents’ parental risky behaviours, namely parental drinking and gambling, as well as parental health status in the last 3 months. Most of these variables were suggested as potential risk factors in the literature [31].

The outcome variable of the study, namely overweight and obesity was assessed using the WHO Global Database of Body Mass Index methods of the calculation of the Body Mass Index of individual respondent. Overweight and obesity were also classified in accordance to the recommended classification methods which was based on an age-and-sex adjusted calculation [32]. For analysis, overweight and obesity were categorised into one group for comparisons with the other group of normal and lighter body weights.

Data were analysed using the Stata V10.0 statistical software program. Since the study was of a cluster sampling design, data were set up with the survey design function utilising the *svy* commands for handling the cluster sampling effect. Bivariate analyses were conducted to examine the unadjusted relationships between variables of interest, the health literacy levels, and overweight and obesity. The majority of variables of interest were categorical or ordinal by nature, except school performance. In terms of the exposure variable, health literacy was categorised into a binary variable of two categories, the low and moderate/high levels, for the ease of analysis. Equality of means among groups was examined using F-tests with adjustments for the cluster sampling design. Chi-squared tests were employed to examine the unadjusted associations between the outcome variable, health literacy, and other potential confounders. Further multivariate analyses were conducted using logistic regression modelling techniques with adjustment for the cluster sampling effect. All significant variables identified in the bivariate analysis and other variables considered as risk factors of overweight or obesity were included in the analysis. A significance level of 5% was used for hypothesis testing. Frist degree interaction terms of potential confounding variables and health literacy were also tested with a significance level of 1%.

## Results

**Figure 1:**
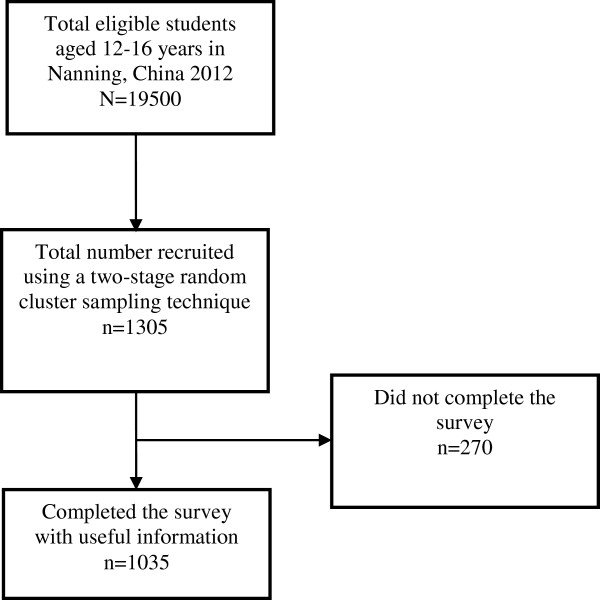
STROBE study conduct and participant flow of the study on health literacy and bodyweight in a 12–16 years old population, Nanning, China, 2012.

The STROBE study conduct and participant flow is outlined in Figure 1. From the total population of 19500 aged 12–16 years adolescents, a sample of 1305 were recruited and all responded to the survey. However, of these 1305 students, 1035 students completed the survey providing usable information. This represented a retention rate of 79.3%. Comparisons on the basic demographics, including age, sex, and classes attended, between the respondents and non-respondents indicated no statistically significant differences. This suggested a representative sample. The characteristics and health literacy of the respondents were summarised in Table 1. The sample consisted of adolescents aged between 12 and 16 years old with slightly more than half aged 14 years or older (n = 722, 55.3%) and a mean age of 14.2 years (s.e. = 0.14). There were slightly more males (59.7%) than females. In terms of demographics, the majority of the respondents were living with their biological parents (n = 1142, 87.5%). There were slightly more non-single child than being the only child in the family (n = 595, 45.6%) with the majority of their parents attaining at least a level of secondary education with about 11% of fathers and 8% of mothers receiving post secondary education levels including university and post graduate education. Less than half (n = 590, 45.2%) of the respondents reported that they did not have a computer at home. For school performance, the average final marks of the last completed school examination was 51.5 (s.e. = 1.37) of a total of 100 marks. About 8% (n = 99) indicate that they were ex-smokers or wereastill smoking at the time of survey, and 551 (42.2%) reported that they had consumed alcohol at least once in the three last months prior to survey. For physical activity involvement, the majority (n = 994, 76.2%) could be classified as inactive.

**Table 1:** Frequency (%), mean (s.e.), and unadjusted associations between health literacy, other socio-demographic or health variables, and overweight/obesity in a 12–16 years old population, Nanning, China (N = 1035)

	**Overweight/Obesity**		Results on association*
	**Yes (n** = **83)**	**No (n** = **1222)**	
**Demographics**			
Sex			
Male	61 (61.5)	646 (52.9)	χ^2^_1_ = 2.30, p = 0.162
Female	32 (38.5)	576 (47.1)	
Age group			
<14	45 (54.2)	538 (44.0)	χ^2^_1_ = 3.27, p = 0.196
≥14	38 (45.8)	684 (56.0)	
Single child			
Yes	41 (49.4)	554 (45.3)	χ^2^_1_ = 0.52, p = 0.404
No	42 (50.6)	668 (54.7)	
**Familial variables**			
Family structure			
Living with both biological parents	73 (88.0)	1069 (87.5)	χ^2^_1_ = 0.02, p = 0.909
Others	10 (12.0)	153 (12.3)	
Father’s education level			
University or above	8 (9.7)	130 (10.6)	χ^2^_3_ = 2.92, p = 0.355
High school/technical college	26 (31.3)	283 (23.2)	
Middle school	35 (42.2)	565 (46.2)	
Primary school or below	14 (16.9)	244 (20.0)	
Mother’s education level			
University or above	5 (6.0)	102 (8.4)	χ^2^_3_ = 2.70, p = 0.514
High school/technical college	21 (25.3)	225 (18.4)	
Middle school	38 (45.8)	603 (49.4)	
Primary school or below	19 (22.9)	292 (23.9)	
Owing a computer at home			
Yes	43(51.8)	547 (44.8)	χ^2^_1_ = 1.56, p = 0.210
No	40 (48.2)	675 (55.2)	
**School performance**	45.8 (1.55)	51.8 (1.43)	F_(1,32)_ = 10.40, p = 0.003
**Health behaviours**			
Smoking status			
Current/Ex-smoker	8 (9.6)	91 (7.5)	χ^2^_1_ = 0.53, p = 0.504
Never	75 (90.4)	1131 (92.5)	
Drank alcohol			
Yes	33 (39.8)	518 (42.4)	χ^2^_1_ = 0.22, p = 0.604
No	50 (60.2)	704 (57.6)	
Physically inactive			
Yes	68 (81.9)	926 (75.8)	χ^2^_1_ = 1.62, p = 0.242
No	15 (18.1)	296 (24.2)	
**Parental health problems**			
Parental drinking problem			
Yes	27 (32.5)	357 (29.2)	χ^2^_1_ = 0.41, p = 0.454
No	56 (67.5)	865 (70.8)	
Parental gambling problem			
Yes	10 (12.1)	176 (14.4)	χ^2^_1_ = 0.35, p = 0.579
No	73 (87.9)	1064 (85.6)	
Parental health problem			
Both parents were ill	7 (8.4)	134 (10.8)	χ^2^_2_ = 7.61, p = 0.009
Either one was ill	12 (14.5)	326 (26.7)	
Both parents were healthy	64 (77.1)	762 (62.4)	
**Low health literacy**			
Yes	56 (67.5)	621 (50.8)	χ^2^_1_ = 8.63, p = 0.017
No	27 (32.5)	601 (49.2)	

*adjusted for cluster sampling effect.

In terms of health conditions and health behaviours of the parents, 384 (29.4%) of the respondents identified that either their father or mother had consumed alcohol to the extent of being drunk in the last 3 months prior to the survey and 186 (14.3%) reported that their parents had gambled in the last 3 months. About a quarter (n = 338) reported that one of their parents was ill in the last 3 months and nearly 11% (n = 141) indicated that both parents were ill in the past 3 months.

For the health literacy levels, 628 (48.1%) respondents could be classified as high, 558 (42.8%) moderate, and 119 (9.1%) were low. In terms of the outcome of the study, slightly more than 6% (n = 83) were classified as overweight (n = 32, 2.9%) or obese (n = 51, 3.9%) according to the WHO classification.

The bivariate relationships between health literacy, demographics, familial variables, school performance, personal risky health behaviour, parental health problems, and overweight or obesity were examined. The results were also summarised in Table 1. As shown, low health literacy was significantly associated with overweight or obesity after adjusting for the cluster sampling effect but not any potential confounding factors (χ^2^_1_ = 8.63, p = 0.017). Of the variables of interest, there were unadjusted associations between school performance, parental health problems, and overweight or obesity (F_(1,32)_ = 10.40, p = 0.003; χ^2^_2_ = 7.61, p = 0.009). These variables with other potential risk factors of overweight or obesity, such as age, sex, and physical inactivity, were included in further analyses as suggested in the literature.

The results obtained from the multivariate logistic regression analyses were presented in Table 2. The results indicated that low health literacy was significantly associated with overweight or obesity among young people (OR = 1. 84, 95% C.I. = 1.13-2.99). After adjusting for potential confounders, young people who were classified as overweight or obese were nearly two times as likely to have low health literacy in comparison those who had a normal body weight. To examine the effect modification between potential confounding variables and health literacy, interaction terms between each potential confounding variable and health literacy was analysed. None of these interaction terms were found significant.

**Table 2:** Association *between health literacy level and overweight/obese in a 12–16 years old population, Nanning, China, 2012

	**OR (95% C.I)**	**Significance**
**Variables**		
Low health literacy	1.84 (1.13-2.99)	t_23_ = 2.46, p = 0.014
Age ≥14 years	0.75 (0.48-1.18)	t_23_ = -1.23, p = 0.219
Being a male	1.18 (0.74-1.90)	t_23_ = 0.70, p = 0.485
School performance	0.99 (0.98-1.00)	t_23_ = -3.00, p = 0.003
Parental health		
Either one was ill	0.47 (0.25-0.89)	t_23_ = -2.31, p = 0.021
Both were ill	0.69 (0.31-1.56)	t_23_ = -0.89, p = 0.374
Physically inactive	1.35 (0.75-2.42)	t_23_ = -0.99, p = 0.321

*Odds Ratios (OR) and 95% confidence interval (CI) from a logistic regression adjusting for all variables presented and for the cluster sampling effect.

## Discussion

This study aims to examine the relationship between low health literacy and overweight or obesity among adolescents in the capital city of Guangxi province of South Western part of China. The results suggest that low health literacy is significantly associated with overweight or obesity. The point estimate prevalence of low health literacy obtained from this study could be compared to that reported in the literature, particularly from the study conducted in Taiwan by Chang et al. [23]. This study found that health literacy levels of 41% of respondents could be classified as high, 49% moderate, and 10% low. In comparison, it shows a slightly higher prevalence of high health literacy levels of 48%, a lower prevalence of moderate level of 43% and a similar prevalence of low level of 9%. However, no statistically significant differences were observed between the two studies (p > 0.05). In the US study, using the category of high level of health literacy as being adequate, the results of this study is also comparable with 52% and 48% reported in the US and the current studies respectively [21]. Hence, these results suggest there is little difference in the prevalence estimate of low health literacy between this particular population of young people in East Asia region and the US. In terms of the prevalence of overweight and obesity, results obtained from this study of about 6% are also comparable to that reported in the literature in China and nearly countries [28,33]. However, for the relationship between low health literacy and overweight or obesity, owning to the lack of study in this area, comparisons of results would be difficult. It is worth noting that the association between low health literacy and overweight/obesity was more significant than that between physical inactivity and overweight/obesity. It is probably due to the imprecise measurement of the physical inactivity in this study.

The association between low health literacy and overweight or obesity could be understood in the wider context of the influencing factors of health literacy. It has been established that health literacy is strongly related to general literacy level, schooling, and school performance in the adult population [34]. Moreover, it has been argued that accessibility to information and educational resources via the Internet may also be a factor contributing to the enhancement of general health literacy [35]. In other words, access to sufficient resources for general education, and more specifically health education, is influential to health literacy levels. For young people who are still under the care of their family, familial resources become one of the influential factors for enhancing their general literacy, as well as the health literacy levels. Children and adolescents who attain a low health literacy level are more likely to come from a low familial resource environment. It would be intrinsic to associate a low familial resource environment with families of a low socioeconomic status that may be characterised with lower parental educational levels, higher proportion of manual labour employment, more involvements of parental unhealthy behaviours such as smoking, alcohol consumption, lack of exercise, and poorer dietary intakes. All these have been identified as risk factors of overweight and obesity. Due to the aforementioned reasons, it would be logical that children and adolescents brought up in such an environment which is not conducive to a better health education and practices would have low health literacy. On the other hand, this sort of environment exposes children and adolescents to risk factors of overweight and obesity. In other words, low health literacy could be one of the many factors, including familial variable and parental health problems, that are interacting and interplaying with each other in the causal pathway of overweight and obesity in young people.

The results obtained from this study have a direct implication on public health policy, as well as school education policy, which could create an impact on the bodyweight problem of young people, particularly in reducing overweight and obesity in adolescents. Since low health literacy is associated with overweight and obesity, it is logical to deduce that enhancing health literacy among adolescents could have an effect in reducing the problem of overweight and obesity in the population. There could be many ways for achieving the goal of enhancing the health literacy levels of young people who have been found of having inadequate health literacy levels. Conducting screening on the health literacy level of all primary and secondary students for identify those who are lacking in the area and provide some remedial enhancement programs could be one. However, this approach is passive, reactive, and piecemeal that may not necessarily generate the expected long-term effect. Another possible approach is to introduce the Healthy School Program, which has been designed in the US and implemented in many countries, in the primary and secondary school system [36]. To further enhance the chance of success in raising the health literacy levels and combating overweight and obesity in the adolescence population, a marriage between the two important global initiatives developed by the WHO and the United Nations Educational, Scientific and Cultural Organization (UNESCO) in the last three decades, namely the Health For All (HFA) and the Education For All (EFA), could be considered [37,38]. Culturally appropriate elements of health education and promotion should be fully integrated in primary and secondary school education curricula as an essential component of school education similar to general literacy and numeracy. It is possible that, under the influence of such education system, the health literacy levels of the future generation could be much enhanced and lead a much healthier life style resulting in a healthier population.

As in all studies, there are strengths and weaknesses in this study. This is a population-based study that includes a random sample of students from two large cities utilising a two-stage cluster random sampling technique. An appropriate statistical analytical approach has been used to adjust for the effect of cluster sampling. The use of a standardised and validated assessment instrument for health literacy minimised some measurement biases. Some potential limitations have also been identified in this study. For example, a cross-sectional study could be considered as an appropriate design for exploring potential risk factors for a condition or disease. However, the evidence provided from such a study can only be considered as associative and it is insufficient to draw any causal inference [39]. This study can be considered as an exploratory study to identify the potential association between low health literacy and overweight or obesity among adolescents. In terms of the outcome measure, namely the classification of overweight and obesity, the classification methods may not have taken into consideration of the pubertal development during the adolescence period of young people. This may introduce a misclassification bias in the study. Some potential confounding factors of childhood and adolescence overweight and obesity, such as diabetes status, parental bodyweight, were not included in this study. A reason for this information not being collected was that children might not have the knowledge of the health status of their parents, particularly their bodyweight. Future studies could be conducted with the inclusion of these variables with data collected from the parents as well as from medical records, as well as employing a better study design such as a longitudinal cohort study that could elucidate whether the association is of a causal nature.

## Conclusions

Results suggested that low health literacy level was associated with many aspects of adolescence health including their body weight. These results have public health implications on an important global problem of adolescence body weight. Enhancing the health literacy should be considered as part of the strategies in combating adolescence weight problem.

## Competing interest

There is no conflict of interest of any kind, both financially and non-financially, that involved in the production of this manuscript by the authors.

## Authors’ contributions

LL initiated the study, designed and developed the study protocol including part of the questionnaire used in the study. He conducted the data analysis with interpretation of results. He also drafted the manuscript. LY conducted the pilot study and carried out field work for the proper study. She supervised the data collection and management process. She assisted in the interpretation of results, provided comments on the manuscript. Both authors read and approved the final manuscript.
